# Biomechanical behavior of atrophic maxillary restorations using the all-on-four concept and long trans-sinus implants: A finite element analysis

**DOI:** 10.34172/joddd.2021.018

**Published:** 2021-05-05

**Authors:** Liliane Pacheco de Carvalho, Alexandre Marcelo de Carvalho, Carlos Eduardo Francischone, Flavia Lucisano Botelho do Amaral, Bruno Salles Sotto-Maior

**Affiliations:** ^1^Department of Prosthodontics, Faculty of Dentistry UNIFAGOC Institute and Research Center, Brazil; ^2^Department of Periodontology, Faculty of Dentistry UNIFAGOC Institute and Research Center, Brazil; ^3^Department of Implantology, Faculty of Dentistry, Dental Research Center of São Leopoldo Mandic, Brazil; ^4^Department of Restorative Dentistry, Faculty of Dentistry, Federal University of Juiz de Fora, Brazil

**Keywords:** All-on-four, Atrophic maxilla, Finite element analysis, Long trans-sinus implants

## Abstract

**Background.** Maxillary bone atrophy with a considerable amount of pneumatization and anterior expansion of the maxillary sinus might be a situation limiting oral rehabilitation with osseointegrated implants. Therefore, the present study aimed to biomechanically evaluate two rehabilitation techniques for maxillary bone atrophy: all-on-four and long trans-sinus implants.

**Methods.** Two three-dimensional models consisting of atrophic maxilla with four implants were simulated. In the M1 model, two axially inserted anterior implants and two tilted implants, 15 mm in length, placed tangential to the maxillary sinus’s anterior wall were used. In the M2 model, two axially inserted anterior implants and two trans-sinus tilted implants, 24 mm in length, were used. For the finite element analysis (FEA), an axial load of 100 N was applied on the entire extension of the prosthesis, simulating a rehabilitation with immediate loading. The peri-implant bone and the infrastructure were analyzed according to the Mohr-Coulomb and Rankine criteria, respectively.

**Results.** The results were similar when the stresses on peri-implant bone were compared: 0.139 and 0.149 for models 1 and 2, respectively. The tension values were lower in the model with trans-sinus implants (27.99 MPa).

**Conclusion.** It was concluded that the two techniques exhibited similar biomechanical behavior, suggesting that the use of long trans-sinus implants could be a new option for atrophic maxilla rehabilitation.

## Introduction

Based on the placement of only four implants for rehabilitating atrophic maxillae, the all-on-four concept has been used and associated with high success rates.^1^ However, in some cases, bone remodeling in the posterior region of the maxilla is so severe that it should be extended to the anterior portion of the maxillary sinus, making it impossible to achieve implant anchorage in the region of the canine abutment, as recommended in the original technique for tilted implants.^2,3^ In these situations, the alternatives for rehabilitation include maxillary sinus lift surgeries using autogenous, homogenous, heterogenous bone, or bone morphogenetic proteins. These techniques have been associated with varied morbidity, depending on the option selected, and demand a waiting time of 4‒12 months for bone repair, making it unfeasible to place implants during the first stage of surgery.^1,4,5^

Thus, the rehabilitation based on anchoring in the remaining bone is an option for the rehabilitation of atrophic jaws with equally high success rates.^1-3^ The use of trans-sinus implants is a treatment option in these cases. The anchoring technique with trans-sinus implants consists of crossing the implant through the maxillary sinus after elevating the sinus membrane and stabilizing it in the anterior wall of the maxillary sinus or the wall of the nasal fossa.^6,7^ Therefore, the implants need to be longer, i.e., 18‒24 mm in length.^8^ The present study aimed to compare the biomechanical performance of total edentulous rehabilitation, the all-on-four concept with tilted implants, and rehabilitation with the use of long trans-sinus implants, subjected to immediate loading in atrophic maxillae, using the finite element analysis (FEA). The null hypothesis indicated no biomechanical difference between the two rehabilitation techniques for edentulous maxilla, all-on-four and long trans-sinus implant concepts.

## Methods

**Figure 1:**
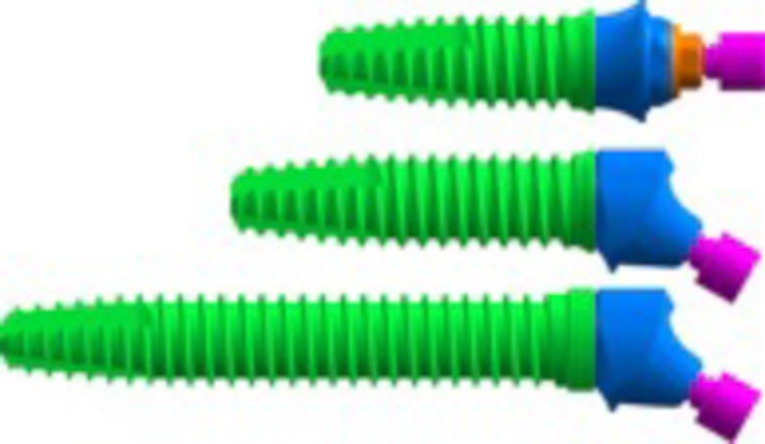
Images of implant models and prosthetic components used in the present study.

Using computer-aided design software (SolidWorks 2019, SolidWorks Corporation), the bone models were constructed based on cone-beam computed tomography cross-sectional images of an edentulous atrophic human maxilla. The images were obtained from the database of the School of Dentistry of the Federal University of Juiz de Fora.^9^ The bone model consisted of medullary bone surrounded by cortical bone with an average thickness of 1 mm, with the aid of the InVesalius and SolidWorks 2019 software. A model of a complete maxillary prosthesis was scanned using a 3D laser scanner (Nextengine HD, Santa Monica, USA) to obtain the three-dimensional geometry of the prosthesis. The models were saved in STL format (Stereolithography, 3D Systems, Rock Hill, USA) for later processing in CAD type SolidWorks 2019 software (Dassault Systems, SolidWorks Corps, USA) to obtain the virtual model of the prosthesis with a metallic bar and dentogingival acrylic resin. The computed models of the implants and prosthetic components (Figure 1) were acquired from the manufacturer (SIN – Sistemas de Implantes Nacional, São Paulo, Brazil).

**Figure 2:**
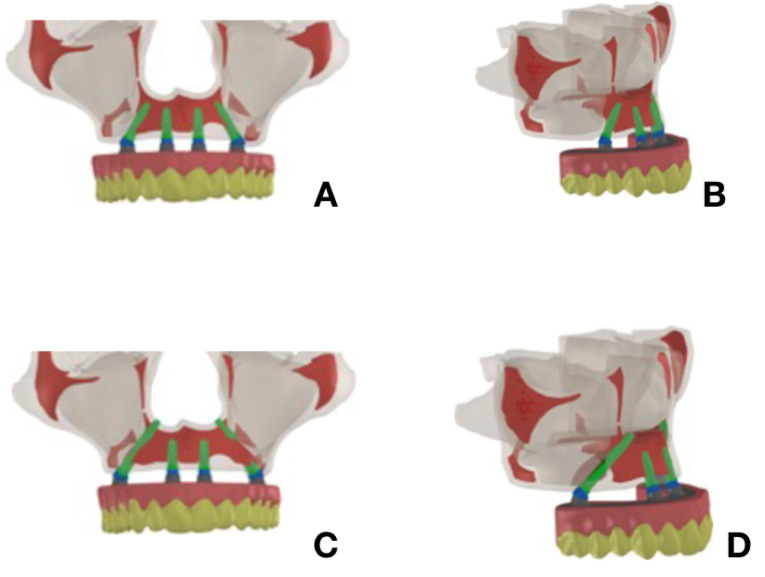
Different views of the final models: M1 (A, B, C), all-on-four, and M2 (C, D, E) ‒ trans-sinus implants.

The models obtained from the tomograph and prosthesis were combined with the models of implants and components to represent a complete, fixed, implant-supported prosthesis with the following characteristics: model M1, simulating treatment according to the all-on-four technique; SW external hexagon implants with a platform 4.1 mm in diameter and a body 3.75 mm in diameter, 11 mm in length (SW HE 3711 SIN- Sistema de Implante Nacional- São Paulo, Brazil) for the anterior implants, and tilted implants 15 mm in length in the posterior region, tangential to the anterior wall of the maxillary sinus (SWHE 3715-SIN –Sistema de Implante Nacional- São Paulo, Brazil) were used. For model M2, simulating treatment with long trans-sinus implants, 11-mm-long anterior implants were placed axially; parallel and tilted implants placed in the posterior region were anchored in the remaining alveolar bone, passing through the maxillary sinus and transfixed in the region of the canine abutment and lateral wall of the nasal fossa.^8^ An access window in the vestibular bone was also modeled, like the type performed in the traditional sinus lift techniques, compatible with the technique analyzed^6^ (Figure 2).

In both models (M1 and M2), the anterior implants placed axially received a straight mini-abutment (MA 3602- SIN –Sistema de Implante Nacional- São Paulo, Brazil), and the distal tilted implants received a mini-abutment placed at an angle of 30º (MAA 3632- SIN –Sistema de Implante Nacional- São Paulo, Brazil).

The models were exported to ANSYS Workbench FEA software (version 14.0; Swanson Analysis) for mesh acquirement and numerical analysis. The mesh was generated with 0.5-mm quadratic tetrahedral elements after convergence analysis (5%) as a refinement process to improve the results’ accuracy and guarantee the mesh quality. Cortical and trabecular bone was assumed to be anisotropic, homogeneous, and linearly elastic. All other materials were considered to be isotropic, homogeneous, and linearly elastic. Mechanical properties of materials were determined from the literature (Table 1). The models were fully constrained in all directions at the nodes on the borders. The models presented several elements ranging from 290.203 to 177.992, and some nodes ranging from 501.571 to 310.143 in each model. The two models were submitted to an axial load of 100 N, with a vector perpendicular to the occlusal plane, applied throughout the entire extent of the prosthesis, simulating immediate loading. The ANSYS® software was used to calculate the values of von Mises stress for cortical and trabecular bone, implants, and prosthetic framework.

**Table 1:** Material attributed to each region of the model and properties of the materials

**Component**	**Young’s modulus (E) (MPa)**		**Shear modulus (G) (MPa)**		**Poisson ratio (δ)**	
Cortical bone	Ex	12600	Gxy	4850	δxu	0.3
	Ey	12600	Gyz	5700	δyz	0.39
	Ez	19400	Gxz	5700	δxz	0.39
Trabecular bone	Ex	1150	Gxy	6800	δxu	0.001
	Ey	2100	Gyz	4340	δyz	0.32
	Ez	1150	Gxz	6800	δxz	0.05
Titanium (implant and abutments)	104000		38800		0.34	
Acrilic	1960		24		0.35	

## Results

Distinct criteria were used to analyze the different materials in the present study due to each material’s inherent characteristics and behavior.

**Figure 3:**
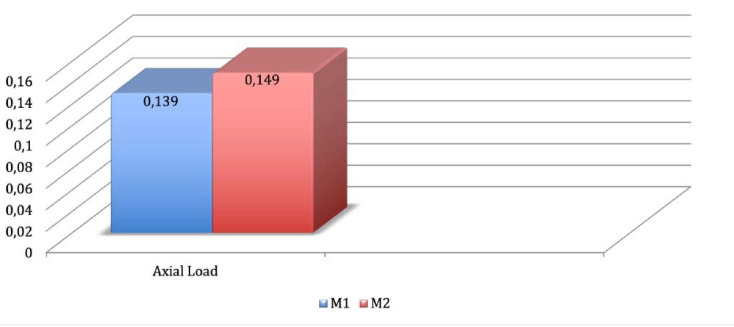
Stresses on the peri-implant bone in immediate loading - Mohr-Coulomb criterion, model M1; all-on-four model M2 ‒ trans-sinus implants.

**Figure 4:**
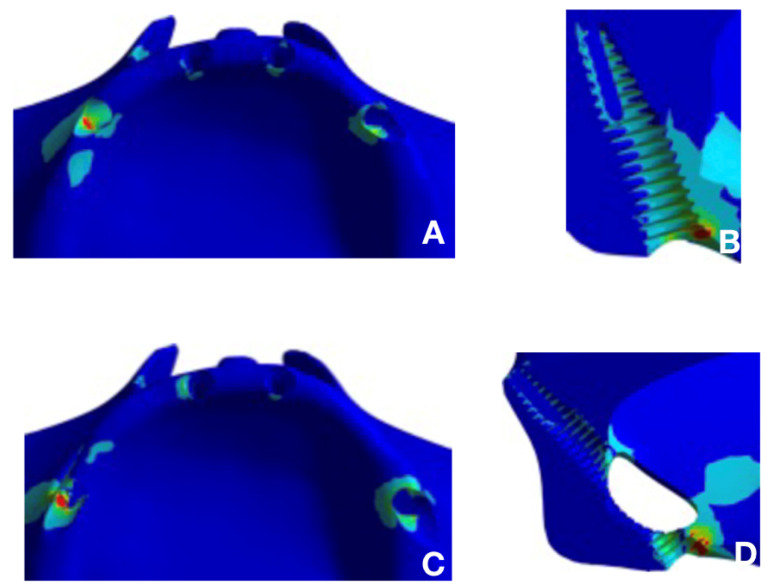
Results on the peri-implant bone under immediate axial load analyzed by the Mohr-Coulomb criterion. M1o Occlusal view, model all-on-four, M1P: palatine view, model all-on-four; M2o occlusal view, model trans-sinus, M2p palatine view, model trans-sinus.

The peri-implant bone was analyzed according to the Mohr-Coulomb criterion. Figures 3 and 4 present the results.

**Figure 5:**
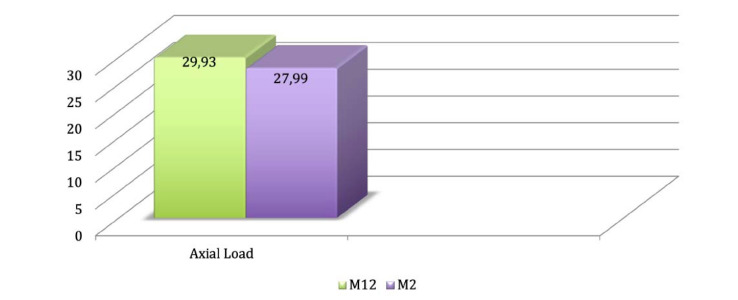
Peaks of traction on the infrastructure according to the Rankine criterion (in MPa) M1 model, all-on-four; M2 model, trans-sinus.

**Figure 6:**
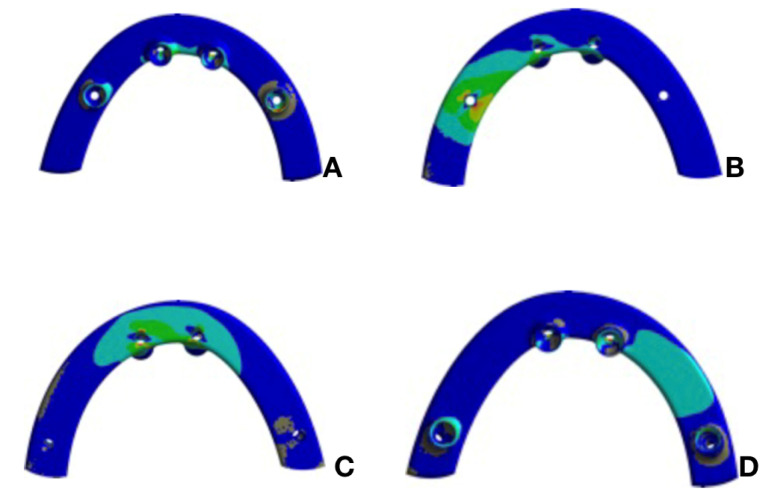
Images of the traction stresses on the prosthesis infrastructure under axial load by the Rankine criterion: M1a, apical view model all-on-four, M1o, occlusal view model all-on-four; M2a, apical view model trans-sinus, M2o, occlusal view model trans-sinus.

The prosthesis infrastructure was analyzed according to the Rankine criterion. Figures 5 and 6 present the results.

## Discussion

Rehabilitation of the atrophic maxilla with the all-on-four concept has been established in the literature.^2,8,9^ The use of trans-sinus implants has also previously been reported by other authors; however, other authors have used conventional implants up to 15-mm long.^6,7,12,13^ The particularity of the present technique lies in the use of extra-long trans-sinus implants up to 24 mm, described in 2017.^8^ However, biomechanical analyses of these rehabilitations are still inadequate in the literature, a factor that prompted the present study.

Given the results, very close stresses on the peri-implant tissues were observed in the two models, with a discrete rise in values in the model with trans-sinus implants. This might be explained by the greater inclination of trans-sinus implants relative to the bone crest, favoring the accumulation of stresses in the peri-implant bone region compared with the all-on-four model. Another fact to consider is the changes in bone insertion. Let us consider the site prepared for the insertion of these implants. It is composed of two anchorage units, with one being represented by the low density and volume and the other by the greater volume and density during a masticatory effort in immediate loading. When a load is applied, there is a tendency to form a gap between the implant and bone of the remaining alveolar ridge, and since it is not yet osseointegrated, the implant tends to undergo slight micromovements within the bone. However, the values obtained in both models were within the acceptable limits for rehabilitation with immediate loading.^14,15^

The present study recommends long implants because they can be inserted in a trans-sinus manner, with apical anchorage on the canine abutment. In addition, this allows a more posterior positioning of its platform. Given the prostheses structure results, a considerable reduction in stress peaks was verified in the model with long trans-sinus implants. This suggested that the emergence of these implants in a posterior position eliminates the extension in the cantilever of the prosthesis, and therefore, increases the polygon of its support, indicating a better mechanical performance of this model. This corroborates the findings of Silva et al,^14^ who observed that the cantilever significantly increased the stress levels on the implant-prosthesis set.

Another fact to consider is that long implants allowed cortical bone insertion in the region of the canine^15-18^ abutment with greater bone density. This is an important condition for primary stability and can give rise to a more favorable distribution of stresses on the prosthesis infrastructure, which could be a decisive factor in the treatment’s longevity. This characteristic overlaps the technique recommended by Malo, in which the implants are tangential to the anterior wall of the sinus and are not considered trans-sinus. Thus, they are only feasible when there is a large volume of remaining alveolar bone in the posterior maxilla. It is a rare finding in the atrophic maxilla.^16,19-21^

In this context, the techniques available for rehabilitating the atrophic maxilla have been efficient, with high success rates. However, in rehabilitations using the immediate loading context, the model with trans-sinus implants showed evidence of a lower risk of bone loss due to mechanical overload.

This biomimetic study was based on the analysis by the 3D FE method. This study’s main limitation is that an axial load simulated static masticatory forces applied to bridge system models. However, the masticatory forces are dynamic and might be directed obliquely to the occlusal surface. It is very challenging to reproduce all the details of natural behavior. Therefore, FEA studies must be used in animal experiments, and longitudinal clinical trials are still necessary.

## Conclusion

Considering the simulations to which the models were submitted, the values of stresses on the peri-implant bone were similar in both models with the conventional all-on-four technique and the model with long trans-sinus implants. However, the values of stress on the prosthesis structure were lower in the model with trans-sinus implants, an essential fact in improving the entire system’s biomechanics. Therefore, long trans-sinus implants might be a new option for the atrophic maxilla rehabilitation with rates that suggest success.

## Authors’ Contributions

LPDC initiated, conceptualized, and supervised the research work. AMDC, CEF, and FLBDA prepared samples and performed the study with the collaboration of LPDC, BSSW, and CEF. All authors have contributed to analyzing the data and writing the manuscript.

## Acknowledgments

The authors would like to thank the UNIFAGOC Institute and Research Center, Brazil, for the authorization of this research.

## Funding

The work has no funding.

## Competing Interests

We declare no conflict of interests.

## Ethics Approval

Not applicable
